# Gang confrontation: The case of Medellin (Colombia)

**DOI:** 10.1371/journal.pone.0225689

**Published:** 2019-12-05

**Authors:** Juan D. Botero, Weisi Guo, Guillem Mosquera, Alan Wilson, Samuel Johnson, Gicela A. Aguirre-Garcia, Leonardo A. Pachon

**Affiliations:** 1 Universidad de Antioquia, Instituto de Física, Medellin, Colombia; 2 University of Warwick, Coventry, United Kingdom; 3 Alan Turing Institute, London, United Kingdom; 4 University of Birmingham, Birmingham, United Kingdom; 5 Centro Nacional de Memoria Histórica, Bogotá, Colombia; 6 Freie Universität Berlin, Lateinamerika-Institut, Berlin, Germany; 7 guane Enterprises, Medellin, Colombia; Arizona State University & Santa Fe Institute, UNITED STATES

## Abstract

Protracted conflict is one of the largest human challenges that have persistently undermined economic and social progress. In recent years, there has been increased emphasis on using statistical and physical science models to better understand both the universal patterns and the underlying mechanics of conflict. Whilst macroscopic power-law fractal patterns have been shown for death-toll in wars and self-excitation models have been shown for roadside ambush attacks, very few works deal with the challenge of complex dynamics between gangs at the intra-city scale. Here, based on contributions to the historical memory of the conflict in Colombia, Medellin’s gang-confrontation-network is presented. It is shown that socio-economic and violence indexes are moderate to highly correlated to the structure of the network. Specifically, the death-toll of conflict is strongly influenced by the leading eigenvalues of the gangs’ conflict adjacency matrix, which serves a proxy for unstable self-excitation from revenge attacks. The distribution of links based on the geographic distance between gangs in confrontation leads to the confirmation that territorial control is a main catalyst of violence and retaliation among gangs. As a first attempt to explore the time evolution of the confrontation network, the Boltzmann-Lotka-Volterra (BLV) dynamic interaction network analysis is applied to quantify the spatial embeddedness of the dynamic relationship between conflicting gangs in Medellin. However, the non-stationary character of the violence in Medellin during the observation period restricts the application of the BLV model and results suggest that more involved and comprehensive models are needed to described the dynamics of Medellin’s armed conflict.

## Introduction

Conflict, in one guise or another, has plagued human progress since historical records began. Protracted conflict is a critical force in stopping societal development and meeting the Millennium Goals. The ability to understand and predict conflict can inform peacekeeping and lead to long-term prosperity [1]. Since the Cold War, conflict has increasingly become subversive, trans-national, trans-genre, and difficult to both define and arrest. Armed violence is often intermixed with illegal smuggling, narcotics, financial crimes and criss-cross several nations. The Colombian conflict is an interesting case of protracted conflict, both due to its complexity in the changing relationship between the governments, paramilitary groups, crime syndicates, and guerrillas; as well as the international attention from the illegal drug trade. The case of Medellin is of particular interest because it is a city that has been suffering the presence of gangs since the 1960s with the rise of the infamous Medellin Cartel. Funding from drug trafficking transformed traditional gang dynamics into violent proxy territorial battles for the cartels. Simultaneously, guerrilla groups, mainly FARC, ELN and EPL, established cooperation pacts with the gangs, increasing their influence and ability to recruit. In the late 90’s and early 00’s the presence of paramilitary groups in the socio-political context of Colombia also allowed those groups to co-opt the gangs in Medellin [2]. After the 2003-2005 demobilization agreement between the paramilitary army and the Colombian government, gangs come back again to the service of the narco-cartels and co-opt local legal economies.

The qualitative arguments on territorial conflict at the intra-city scale have been well understood over the decades through extensive ground-level studies by the *Instituto de Estudios Politicos– UdeA*, *Instituto Popular de Cultura*, *Centro de Analisis Politico—Universidad EAFIT* and recently the *Centro Nacional de Memoria Histoórica* (CNMH). However, there lacks a unified quantitative model which can both describe the chain of historical conflict events, as well as potentially forecast future conflict dynamics.

### Literature review

Conflict prediction can generally be divided between data-driven statistical methods and causal reasoning [1]. In data-driven methods, statistical trends (e.g. cycles, power-laws [3], spatial-temporal processes [4]) are used to guide prediction. Recently, the availability of high spatial resolution data allowed researchers to have significant impact in the field [5, 6] and show that the statistical patterns are significant [7–10], and can have self-excitation behaviour (i.e., Hawkes process) [11]. However, the accuracy of such models is either confined to the aggregate scale or fine-tuned to work in a highly specific context. Furthermore, the low-dimensional model parameters often do not naturally reflect the multi-dimensional causal factors. On the other hand, causal reasoning is used to combine domain expertise and real-time knowledge to predict violence [12]. Groups of experts have been shown to be effective in reducing bias and surveys of experts to quantify risk predicts general trends well [13].

Alternative Agent-Based Model (ABM) approaches are on the other hand able to test hypotheses and causal mechanisms such as policy interventions. Many attempts have been made to create mechanisms that explain conflict using interacting agents, including: clash of cultures [14], distribution of political responsibility, technology transfer [15], foreign aid fluctuations, and deterioration of the natural environment. However, their complexity and data dependency means that a universal ABM is absent.

Scalable ground census using natural language processing also works well when a curated target-specific learning [16]. However, such approaches do not integrate the growing data collection (e.g. ACLED, UCDP, GTD) and data science capabilities. Recently, moving beyond logistic regression, higher dimensional machine learning approaches that combine multiple causal factors and big data have been used to predict violence. Techniques such as Random Forest are able to indicate the relative importance of different factors but lack the mechanical insight to indicate why [17]. Furthermore, over-fitting and catastrophic forgetting are critical issues which will prevent the method from predicting unexpected new events. Indeed, even advanced deep learning techniques are likely to predict self-regressive behaviour (e.g., protracted war), but not new events [1].

The third modelling category belongs to interaction dynamics, which attempt to model the key relationship dynamics between actors. As interactions underpins the fabric of human society across multiple population scales, methods such as the entropy-maximising Boltzmann-Lotka-Volterra (BLV) spatial interaction model can describe the projected flow of threat or influence between adjacent population groups [18]. Such models have been used to model ancient conflicts [19] and predict the likelihood of new ones [20].

The Boltzmann-Lotka-Volterra (BLV) model feeds from, e.g., the gravity-based principle [21, 22] in the social sciences that provided a formal strategy to assets the effect geographic distance on connectivity of spatial networks dynamics and human behaviour [21–26]. The idea that the likelihood of a relationship (e.g., social or economic) is inversely proportional to the physical distance between two entities (Refs. [21, 22]). In economic geography, the gravity model was used to explain migration flows between countries, regions, or cities [24], and showed that movement of people between cities is proportional to the product of their population size and inversely proportional to the square of the distance between them. In the context of international economics, the gravity model of trade predicts trade- flow volumes and capital flows between two units to be directly proportional to the economic sizes of the units (using GDP data) and inversely proportional to the distance between them [23].

In the context of corporate competition, spatial network analysis was utilized to show [26] that the spatial locations of firms are positively correlated with the population density, and that firm competition networks are governed by cumulative advantage rules and geographic distance (which is equivalent to the BLV). In the contexts of civil unrest and riots [25, 27], it has been shown, both theoretically and empirically that social unrest dynamics is based on the hypothesis that widespread unrest arises from internal processes of positive feedback and cascading effects in the form of contagion and social diffusion over spatially interdependent regions connected through social and mass communication networks. So that social instability can be considered as a spatial epidemics phenomenon, similar to other spatially extended dynamical systems in the physical and biological sciences, such as earthquakes, forest fires, and epidemics. This perspective was confirmed by modelling the 2005 French riots using spatial epidemiological models [27]. Here we extend these ideas to the case of urban paramilitary groups in their hegemonization process in Medellin, Colombia.

### Contribution

The gang confrontation network, over twenty-years of intense conflict, of Medellin is presented. It is shown that the violence escalation is highly correlated with socio-economical indexes like the Gini Coefficient, Human Right Violations, Homicide Rate and Unemployment Rate. A high correlation between the structure of the gang confrontation network and the escalation of conflict in Medellin is presented. The collected data was analysed under the light of network theory and models from complex systems were employed to simulate the structure of the network. Specifically, application of the Boltzmann-Lotka-Vollterra model confirmed that the conflict network of gangs in Medellin is spatial and therefore in strongly driven by territory control. However, results also suggest that more involved and comprehensive models are needed to described the dynamics of Medellin’s armed conflict.

This document is organized into four sections. The Introduction reviews the context of the gangs in Medellin and the mathematical models implemented previously in a similar context. Materials and Methods Section discusses the data sources and presents the dynamic analysis of networks and the BLV formalism. Results and Discussion Section covers the main results obtained from the gang’s conflict network, the relation between socio-economic and network properties with the escalation of violence and the simulations obtained after the implementation of BLV methodology. Finally, in Conclusions Section, the results are summarized.

## Materials and methods

### Data sources

After the demobilization of paramilitary groups, Colombia government created the *Centro Nacional de Memoria Histórica*–CNMH (National Center for Historical Memory) to collect and process the contributions from demobilized people to the historical memory of the conflict. The CNMH reconstructed the memory of Medellin conflict in the Law 1424 Historical Memory Report on the Bloque Metro, Bloque Cacique Nutibara and Bloque Heroes de Granda that are paramilitary structures that operated in Medellin. In the framework of that report, information on the ego of gangs and their relationships were identified and processed for six well defined periods of time: (i) previous to the incursion of the Bloque Metro, ca. 1995-2000; (ii) during the presence of the Bloque Metro, ca. 2000-2002; (iii) during the war of the Bloque Metro and the Bloque Cacique Nutibara that annihilated the Bloque Metro, ca. 2002; (iv) during the presence of the Bloque Cacique Nutibara that demobilized in 2003, ca. 2003 (v) during the presence of the Bloque Heroes de Granada, ca. 2003-2008 that demobilized in 2005 and (vi) during the Demobilization, Disarmament and Reintegration (DDR) to civil life period, ca. 2008-2014.

Specifically, the ego of the gang comprises information on the participation in paramilitary groups, the illegal economies they controlled, the area of influence and an approximated number of members. Due to the confidential character of the information, gangs were labeled with a unique code with no more information than a label for the administrative zone of the city where they operated and a random number, e.g., CE026 denotes a gang in the Center-Eastern zone (CE) and 026 is a random number associated to that gang in that zone. As expected, gangs come from and operate in the most conflicted zones of Medellin’s, namely, Center-Eastern (CE), North-Eastern (NE), Center-Western (CW) and North-Western (NW) zones. The information on the type of (i) confrontation, (ii) collaboration among gangs, (iii) godfathership, (iv) subservience and (v) types of confrontation and collaboration between gangs and State Agencies were registered for completeness. The data set was complemented with information from local media. For the present analysis, in the framework of the Cooperation Agreement between CNMH and Universidad de Antioquia (UdeA), only the dataset associated to confrontations is utilized.

Confrontations are characterized by successive and systematic acts of direct violence, or escalated during periods of latency and expressed through one or successive acts of direct violence of different types between gangs over a period. Violence actions include shootings, harassment, homicides, forced displacement, threats; among a long list of violent actions that involve gang members and/or civil population of the territory under their illegal armed control. Therefore, confrontations refer to the existence of enmity relationships or armed antagonism between gangs and not to a specific violent act. Medellin’s armed-conflict-nature suggested the formulation of three confrontation main categories: (1) direct conflict between two gangs acting by themselves, (2) conflict between a gang pertaining to a paramilitary or guerrilla group and a gang acting by itself and (3) conflict between gangs pertaining to different paramilitary or guerrilla groups. The reasons for igniting a particular confrontation were registered as: (A) Interpersonal, (B) Territory defense and control, (C) Control of micro economies, (D) Control of macro economies, (E) Loyalty to a macro structure, (F) Counterinsurgency, (G) Self-defense and/or (H) Drug trafficking.

For instance, the confrontation between CE026 and CN032 may be characterized, e.g., as 1A, 1B. From the information collected, five directed and weighted networks were constructed with the nodes being the gangs and State Agencies. For the confrontation network, edges start in the node that ignites the confrontation and end at the nodes upon which the action rests. The weight of the edges corresponds to the number of confrontation codes needed to characterized the type of confrontation between two gangs. In the example above, if CE026 ignited the confrontation against CN032 and the confrontation was characterized as 1A, 1B (two confrontation codes); then, the edge starts in CE026, ends in CN032 and has weight 2. The networks for each period were constructed, as previously done, e.g., in Ref. [26], using the “snowball sampling” [28].

The CNMH-UdeA collaboration identified 671 gangs in the city across the six periods. During the observation periods, some gangs were annihilated and new gangs were created so that nodes may change from period to period. Of the total of gangs identified in the city, information was found only for 317 of them. The reasons for the lack of information, in particular, for the last two periods was the apprehensiveness of the demobilized people to contribute with information on illegal activities once they were officially reintegrated into the legal-civil society. Moreover, due to the intricate nature of the conflict, it was not always possible to infer the direction of the edges; in these cases, undirected links were utilized. The data was independently collected and processed by seven social scientists from ca. 70% of the officially demobilized people that operated in Medellin and under the supervision of the lead team of CNMH and UdeA. The data was then shared and confronted by all the members of the collaboration to agree, e.g., on the number of confrontation codes needed to characterize each confrontation and to deliver a first unified version of the network. A comprehensive quantitative analysis of the networks and gangs information is in progress.

#### Data availability

The information about the number of violence acts are taken from the *Observatorio Nacional de Memoria y Conflicto* of CNMH and accounts for information on infringements of International Humanitarian Law, namely, war actions, selective assassination, terrorist attack, damage to civilian property, enforced disappearance, massacre, recruitment, kidnapping, sexual violence. The Gini coefficient, Unemployment and Homicide Rates were compiled by the authors from open data provided by the *Departamento Administrativo Nacional de Estadistica of Colombia* (DANE). The datasets generated and/or analysed during the current study are attached as supporting information (see, S1 Table, S2 and S3 Scripts).

### Spectral analysis: A dynamic analysis motivation

The presence of paramilitary structures in Medellin can be conceived as a dynamical network with the main distinctive stages depicted by the six periods described above. However, the annihilation and emergence of dominant structures of very different character, namely, anti-insurgent (Bloque Metro), narco-paramilitary (Bloque Cacique Nutibara) and political-paramilitary (Bloque Heroes de Granada) suggest a separate analysis to uncover similarities and differences in their *modus operandi*. Thus, instead of directly addressing the dynamics of the network across every stage, the global properties of each network are characterised below by means of a spectral analysis of the confrontation adjacency matrix.

The eigenvalues of the adjacency matrix can be clearly related to the dynamics of the conflict. In doing so, define a state vector ***P*** with components {*p*_*i*_}. The linearised dynamics satisfy ${\dot{p}}_{i}(t)=-p_{i}(t)+\sum_{j}a_{ij}p_{j}(t),$ where A = *a_ij_* is the adjacency matrix. *a*_*ij*_ contains the information about existence of confrontation among the *i*^th^ and *j*^th^ nodes (gangs) whereas *p*_*k*_ quantifies the intensity of the violence exerted or suffered by the *k*^th^ node. In matrix notation, $\dot{P}\left(t\right)=-P\left(t\right)+P\left(t\right){\text{A}}^{⊤}$. To decouple this set of equations, note that it can be written as [29]

$$\begin{array}{c} \dot{x}\left(t\right)=-x\left(t\right)+\lambda x\left(t\right), \end{array}$$ (1)

where **x** = ***P*** e and λ and e being the eigenvalues and eigenvectors matrix of A^⊤^, respectively. Since networks considered here are undirected, i.e., A is symmetric and real, then its eigenvalues are real. Therefore, the system described by the differential Eq (1) reaches a stable regime only for λ < 1; for other values of λ, solutions diverge in the long-time regime. Moreover, the Perron-Frobenius theorem [30] guarantees that A will have a unique positive leading eigenvalue λ_L_ and the dynamics of the system will be mainly governed by this dominant eigenvalue of A. The second largest eigenvalue $\lambda_{L_{2}}$ can be interpreted as a second order correction in the stability analysis rate of convergence to equilibrium distribution.

The war rules of each period are assumed to be encoded in the adjacency matrix. Thus, the assumption here is that during each period the characteristics of the linearised dynamics are governed by the adjacency matrix. This assumption, not verified here due to the lack of information for every period, is then utilized to compare the global properties between all stages of Medellin’s conflict.

### Boltzmann-Lotka-Volterra models for conflict networks

As a first attempt to find an analytical model for describing the confrontation network in Medellin, consider the Boltzmann-Lotka-Volterra (BLV) that have been widely used to model spatial networks [18, 31–33]. This approach applies when the external dynamics that may trigger conflicts (i.e., climate change and drought) are quasi-static over the time period of a few decades [34]. Being BLV method a benchmark model in the field, it is relevant to see how it performs here although since the data may be unlikely to be stationary. However, this provides a baseline for opening new lines of research that can improve on our initial findings.

The main goal of the BLV formalism is to merge two well-known models in science: the maximisation of entropy proposed by Boltzmann and a competition model also known as predator-prey model proposed by Lotka and Volterra. The target of this formalism is to predict the values of the ties among the nodes that constitute the network, i.e., to generate the adjacency matrix. The values of *a*_*ij*_ predicted by the theory will be bounded between 0 and 1, thus generating a weighted network that should maximise the entropy functional *S* = −∑_*ij*_ *a*_*ij*_ log *a*_*ij*_ with ∑_*ij*_ *a*_*ij*_ *d*_*ij*_ = *C* and ∑_*ij*_(*a*_*ij*_ log *p*_*i*_ + *a*_*ij*_ log *p*_*j*_) = *B*. Here, *d*_*ij*_ is the distance between the nodes *i* and *j* and *p*_*i*_ is the benefit associated to the *i*^th^-node, *C* and *B* are constants that can be understood as the total spatial cost and the total benefit, respectively. Note that the BLV formalism is, therefore, a methodology to find the optimal solution of a cost-benefit problem.

By solving the constrained optimisation problem established above, the weight of the links between the nodes, as a generalisation of the Boltzmann probability distribution, can be obtained from

$$\begin{array}{c} a_{ij}=\frac{{\left(p_{i}p_{j}\right)}^{\alpha }e^{-\beta d_{ij}}}{\sum_{ij}{\left(p_{i}p_{j}\right)}^{\alpha }e^{-\beta d_{ij}}}, \end{array}$$ (2)

where *α* and *β* are the Lagrange multipliers required for solving the constrained optimisation problem. A similar approach, but in the context of corporate competition, was performed in Ref. [26] by Braha *et al*. They proposed a model combining preferential attachment and geographic distance effects, where the probability of competition between firms will be proportional to ${\left(k_{i}k_{j}\right)}^{\gamma }d_{ij}^{\delta }$. The key idea is that the physical distance between nodes strongly determines the benefits and costs of transport and communication [26], which then have significant importance in the context of gang war and company competition.

With no loss of generality, *α* is set to 1 in Eq (2) and the degree-preference model is assumed, i.e., *p*_*i*_ → *k*_*i*_, where *k*_*i*_ is the degree of the *i*^th^-node of the network obtained from the data. Hence, the links can be written as

$$\begin{array}{c} a_{ij}=\frac{\left(k_{i}k_{j}\right)e^{-\beta d_{ij}}}{\sum_{ij}\left(k_{i}k_{j}\right)e^{-\beta d_{ij}}}. \end{array}$$ (3)

The goal is to obtain the optimal value of *β* in Eq (3) that best fits the confrontation network reconstructed from the collected data. The predicted network is generated as follows: (i) the weighted edges are generated according to Eq (3); (ii) only the *N*_ed_ biggest values are selected– *N*_ed_ is the number of edges in the real network–; and (iii) the weights are set to 1 and the rest of them equal to 0. This was done to assure that both, the real and generated adjacency matrices, have the same number of links and are binary matrices.

The accuracy of the adjacency matrix obtained from the model is measured by the distance, in the space of matrix, to the the adjacency matrix reconstructed from the collected data. The distance can be calculated, e.g., by means of the Frobenius distance $D_{F}=\sqrt{\mathrm{tr}\left(A^{r}-A^{m}\right){\left(A^{r}-A^{m}\right)}^{T}}$, multiplying distance $D_{M}=1-N^{-1}\sum_{ij}A_{ij}^{r}A_{ij}^{m}$ or the subtraction distance $D_{S}=N^{-1}\sum_{ij}\left|A_{ij}^{r}-A_{ij}^{m}\right|$. *A*^r^ is the adjacency matrix reconstructed from demobilized people contributions and *A*_m_ the adjacency matrix obtained from the model. Finding the value of *β* in Eq (3) that minimises the matrix distance allows for reconstructing the conflict interaction network using the BLV methodology.

## Results and discussion

### General description of the confrontation network

**Fig 1:**
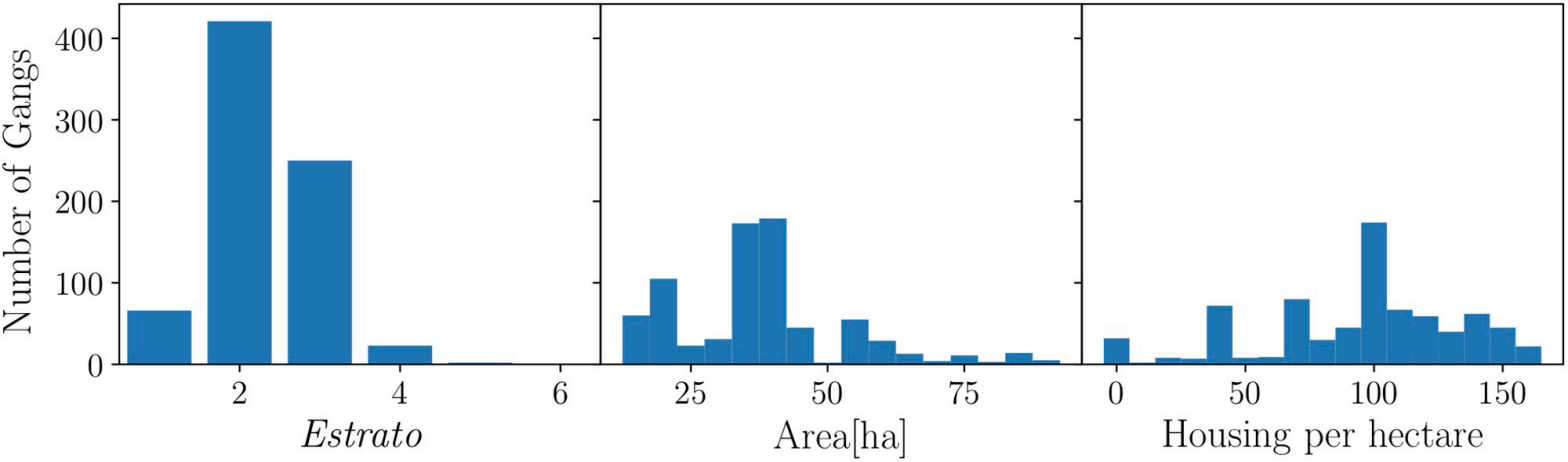
Left panel: Number of gangs per *Estrato*. Central panel: Comparison between the number of gangs and the size area per neighboorhood. Right panel: Comparison between the number of gangs and the density of housing per neighboorhood.

The distribution of gangs in the city is sociologically understood in terms of the late colonisation of the city, mainly, by victims of forced displacement from the countryside of Antioquia department. Moreover, In Colombia, cities are divided into *Estratos* from one to six. Citizens who live in the lowest *Estratos*– one, two and three– have reduced-fares for public services (water, gas, electricity). Citizens who live in the highest *Estratos*– five and six– covers the reduction for the lowest *Estratos*. *Estrato* four corresponds to middle class, citizens who pay what they consume. To quantitatively justify the distribution of gangs in the city, it has been commonly assumed that the distribution and density of gangs obey geo-economics criteria. To test the hypothesis above, Fig 1 presents the number of gangs as a function of (i) the *Estrato* of the neighboorhood, (ii) the area of the neighboorhood and (iii) the housing density per hectare. The main presence of gangs in the lowest *Estratos* can be socio-economically understood. However, the dependence on the area of the neighboorhood and the housing density per hectare suggests that there is no trivial explanation for the presence of gangs in different zones of the city. For the case of corporate competition, in Ref. [26], it was demonstrated that the spatial locations of firms are positively correlated with population density. Although this result is intuitively clear, it cannot be generalized straightforwardly to the case of gangs in Medellin because of its topography: a small valley that accommodates people homogeneously so that different the zones of the cities are fairly equally populated [Colombia official census 2005 by *Departamento Administrativo Nacional de Estadistica of Colombia* (DANE)’s]. Therefore, to fully understand the gang phenomenon in Medellin from a geographic perspective, a further socio-demography analysis is needed and will be conducted elsewhere.

#### Tolopogy of confrontation network

Since it was not possible to clearly identify the area of operation of all gangs, below, two datasets are considered: (i) The weighted full network that contains information of 317 gangs. For this case, the 1995-2000 network comprises 186 nodes and 277 links, in shorthand notation (186:277), the 2000-2002 network comprises (127:148) whereas the 2002 network (91:122) and the 2003, 2003-2008 and 2008-2014 networks have (83:131), (40:48) and (80:119), respectively. (ii) The unweighted geo-referenced network comprises the gangs for which it was possible to collect georeferencing information; additionally, this network considers no difference between having one or several reasons to get into conflict. The topology analysis is performed only for the full network whereas the subsequent analysis is comparatively presented, when possible, for both networks. Information needed to reproduce the results presented here is provided in the Supplementary Information.

**Fig 2:**
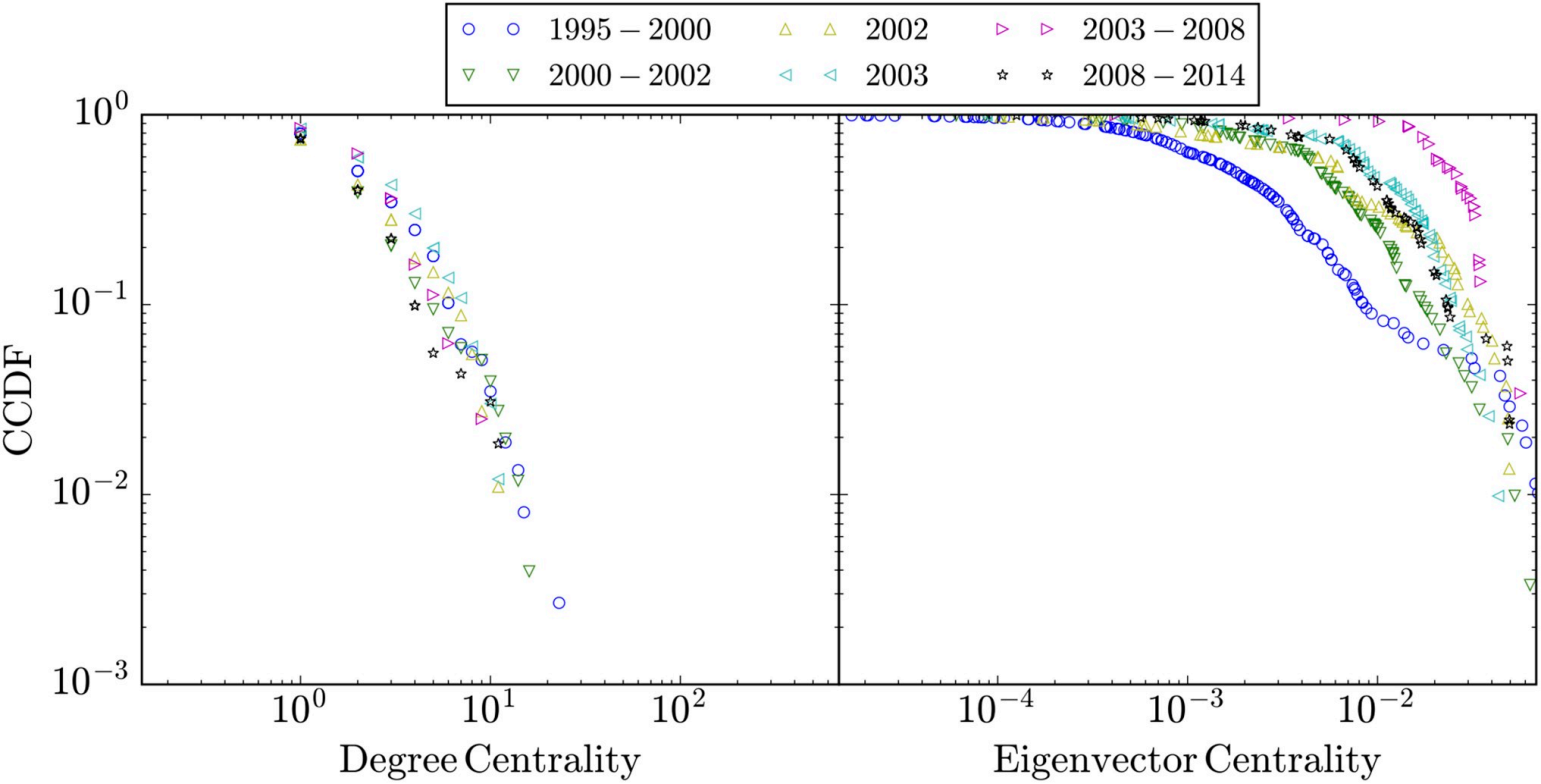
The log-log plots of the Complementary Cumulative Distribution Function (CCDF) of nodes centralities. The left hand side panel corresponds to the degree centrality measure whereas the right hand side panel corresponds to the eigenvector centrality measure.

For the six periods introduced above and under the assumption that the full network is undirected, Fig 2 depicts the cumulative probability function for two different centrality measures, namely, degree and eigenvector centralities. The Complementary Cumulative Distribution Function (CCDF) for the six periods was fitted to five known distribution functions, namely, stretched exponential, exponential, power, power with cutoff and LogNormal functions. Results are displayed in Table 1. Based on the Bayesian Information Criterion (BIC), it is concluded that the CCDF can be accurately described by an exponential model ${\text{e}}^{-\lambda_{\text{c}}x}$. Table 2 presents the relevant parameter information for the fit.

**Table 1:** Bayesian Information Criterion (BIC) for various fitting models of the centrality CCFCs. *k*_c_ is calculate from $\int_{x_{\text{min}}}^{\infty }dxk_{c}f\left(x\right)=1$ with *x*_min_ the the lower bound of the range of possible values that the random variable can attain. BIC is defined as *k* ln *n* − 2 ln *L* with *k* being the number of parameters estimated by the model, *n* number of data points in *x*, *x* the observed data and *L* the maximised value of the likelihood function of the model. The Model with the lowest BIC is preferred.

BIC for Degree Centrality Distributions Models					
Network	Stretched Exp.	Exp.	Power	Power Cutoff	LogNormal
	$k_{c}x^{\beta_{c}-1}e^{-\lambda_{c}x^{\beta_{c}}}$	$k_{c}{\text{e}}^{-\lambda_{\text{c}}x}$	$k_{c}x^{-\gamma_{c}}$	$k_{c}x^{-\gamma_{c}}e^{-\lambda_{c}x}$	$k_{c}x^{-1}e^{-\frac{{\left(\text{ln}\;x-\mu \right)}^{2}}{2\sigma^{2}}}$
1995-2000	-576.259	-**581.441**	4729.93	-579.259	341.53
2000-2002	-276.601	-**281.462**	210.807	-277.87	231.971
2002	-293.043	-**297.582**	153.215	-293.789	169.178
2003	-238.784	-**243.113**	138.868	-239.101	157.503
2003-2008	-77.3534	-**81.0855**	72.8323	-77.4182	77.4182
2008-2014	-180.69	-**185.081**	136.377	-181.201	151.169
BIC for Eigenvector Centrality Distributions Models					
Network	Stretched Exp.	Exp.	Power	Power Cutoff	LogNormal
1995-2000	-768.546	-763.403	4776.1	**-770.574**	364.752
2000-2002	-602.729	-**606.312**	3288.79	-602.956	233.429
2002	-290.716	-257.489	98.2406	-**293.994**	176.977
2003	-298.711	-**301.261**	82.0277	-299.546	159.457
2003-2008	-39.6239	-**40.7717**	39.4389	-40.4037	83.023
2008-2014	-298.088	-292.277	71.6443	-**299.771**	149.643

**Table 2:** Fitting parameters of the CCDF in Fig 2 to the exponential function ${\text{e}}^{-\lambda_{\text{c}}x}$.

Network	Degree Centrality		Eigenvector Centrality	
	λ_c_		λ_c_	
	EstValue	StError	EstValue	StError
1995-2000	0.315704	0.00399511	365.557	3.38807
2000-2002	0.39655	0.00914728	136.155	0.967632
2002	0.365193	0.00616567	105.908	2.65042
2003	0.2711	0.00548173	73.0748	1.06943
2003-2008	0.300517	0.0127068	29.7294	1.95771
2008-2014	0.401977	0.0108252	82.2071	1.18253

**Fig 3:**
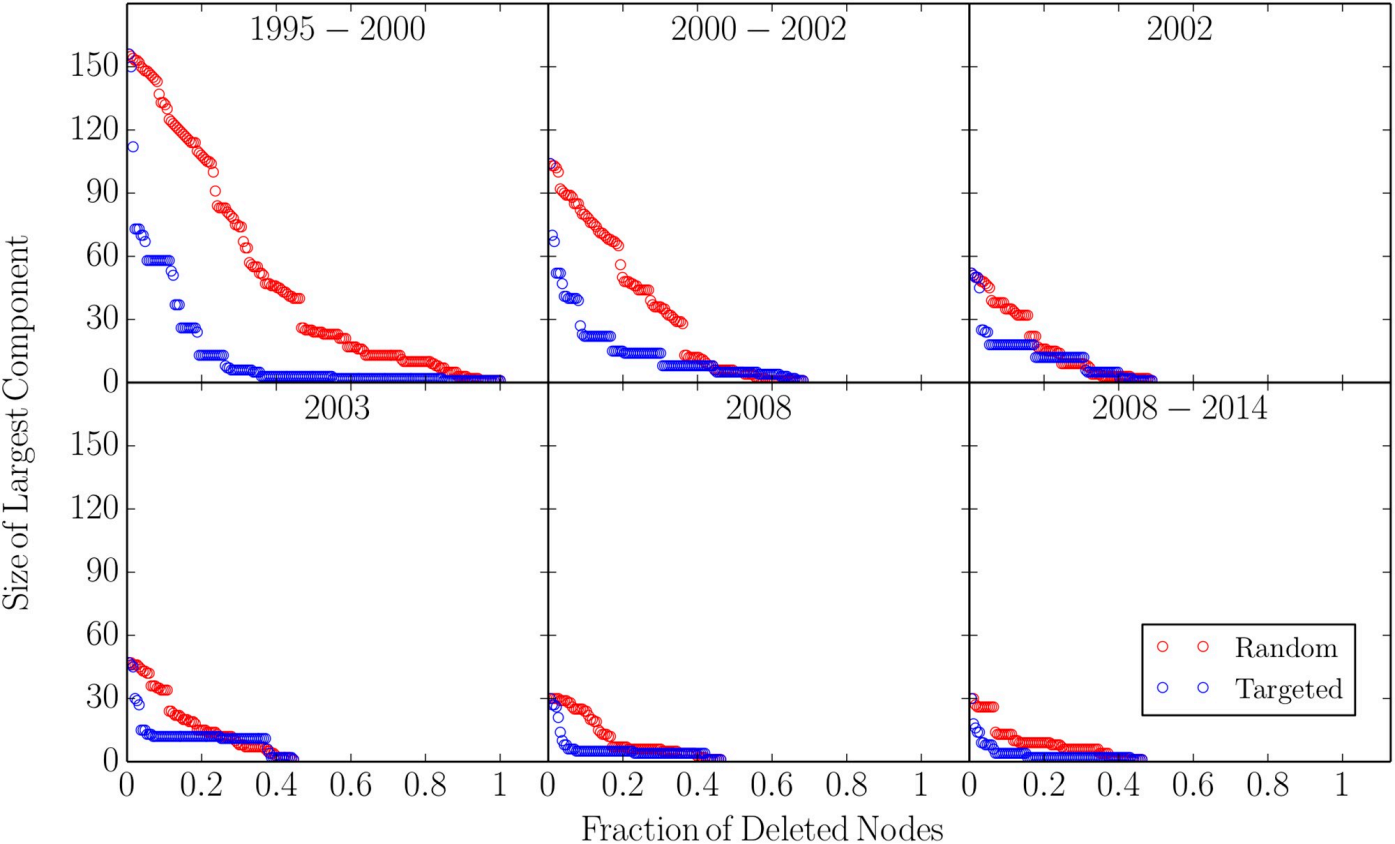
Robustness of the confrontation network, as demonstrated by the deletion of nodes. Red open circles (upper) shows the size of the largest strong component surviving as nodes are randomly deleted; Blue open circles (lower) shows the size of the largest component as nodes are deleted in order of decreasing degree centrality.

As in Ref. [26], the robustness of the confrontation network is analysed below in terms of the size of the largest of the network after (i) removing randomly nodes of the network or (ii) removing the nodes in order of decreasing degree centrality. A network is consider fragile if after excising a single node it falls apart into many small pieces. The global behaviour observed in Fig 3 is that networks are resilient against removing random nodes, but weak against the targeted deletion of high–degree nodes. Thus, the confrontation network of gangs in Medellin is fragile against removing highly connected nodes.

### Socio-economic context of Medellin’s gang confrontation network

In terms of violence activity, there are two recent significant peaks in Medellin: one in 2002 and the other in 2010, which split the history into three significant periods. The first period (1995-2002) is related to the incursion of the paramilitary army in the city that lead to an upsurge in violence among the left- and right-wing gangs. This period ended with the peace process of the Colombian government with the paramilitary groups, a process that concluded with the demobilisation of 31.671 people in the whole country and corresponded to a significant reduction of Human Rights Violations until 2008. Between 2009-2010 other illegal armed groups such as El Clan del Golfo and La Oficina vied to take control over the illicit business in the city which led to the second wave of recrudescence of violence.

**Fig 4:**
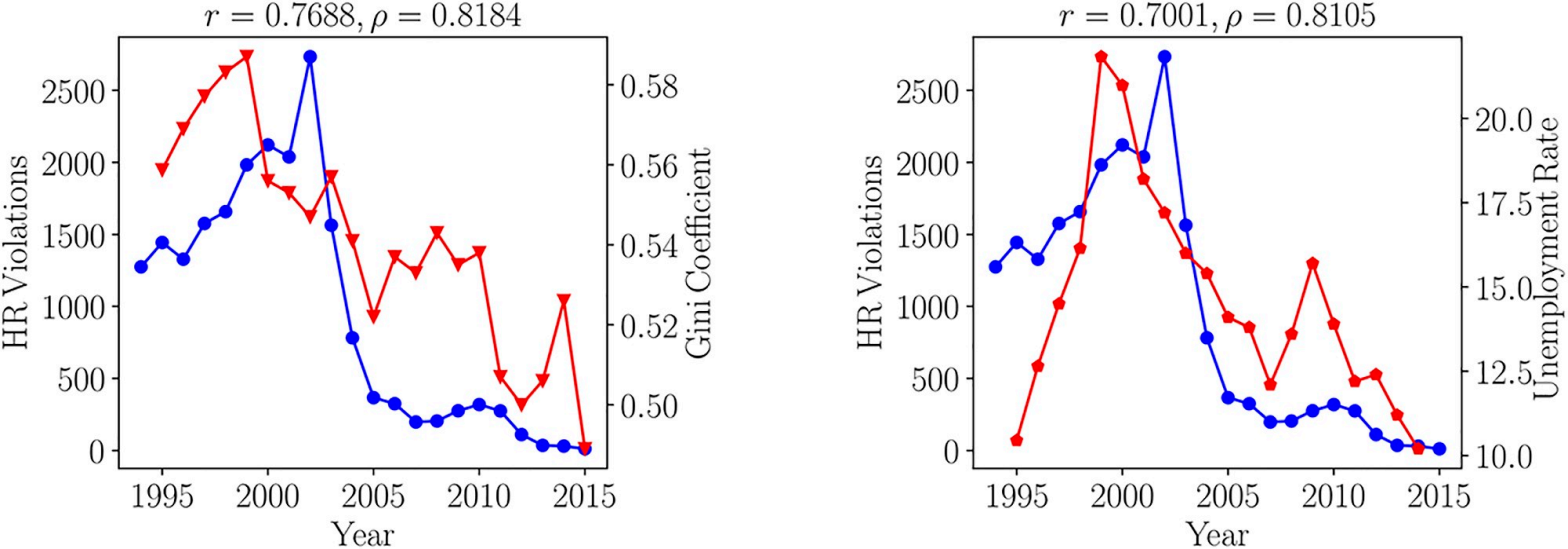
BLUE: Time series of the number of Human Rights Violations (HR Violations) in Medellin during the years 1995-2015. RED: (left panel) Gini Coefficient for Medellin and (right panel) Unemployment Rate in Medellin between 1995-2015.

Fig 4 depicts the time series of the numbers of violations of Human Rights in Medellin with two significant socio-economic factors: the Unemployment Rate (left panel) and the Gini Coefficient (right panel). Linear, measured by the *r*-Pearson coefficient, as well as non-linear, measured by the *ρ*-Spearman coefficient, correlations are displayed in each panel. Fig 4 shows that the Unemployment Rate (UR) and the Gini Coefficient are highly correlated, with the number of Human Rights Violations (HR Violations).

For the data in Fig 4, the Unemployment Rate extracted from the reports of the *Departamento Administrativo Nacional de Estadistica of Colombia* (DANE)’s. For 1995 to 2000, the annual Unemployment Rate was calculated as the arithmetic average of the quarterly reported unemployment rate. For 2001 to 2015, the annual annual Unemployment Rate was literally extracted from DANE’s reports. As to the Gini coefficient, DANE started reporting it only in 2002. In 2005, it changed its methodology and did no report data for 2006 and 2007. The Gini coefficient time-series was completed as follows: data for 2001, 2006 and 2007 was calculated by using a linear regressor from the reported data (see, S1 Table for details) and the data from 1995 to 2000 was obtained from reports of the World Bank for Colombia, which was additionally supplemented by a linear interpolation to get an approximate coefficient for 1996,1997 and 1998.

Since not all Human Rights Violations can be exclusively associated to the gang conflict, the context analysis is complemented with the Homicide Rate per 100,000 inhabitants, the category that is mainly associated to the gang conflict. These four variables comprises a fully-connected and strongly correlated network (see below), thus confirming the correlation between violence and socio-economic factors. Focus here is on finding how the structure of the conflict network affects the behaviour of the gangs and violence indexes in Medellin. Specifically, how the number of gangs *N*_g_, the leading λ_L_ and second leading $\lambda_{L_{2}}$ eigenvalues of the adjacency matrix correlate with the socio-economic and violence indexes.

**Fig 5:**
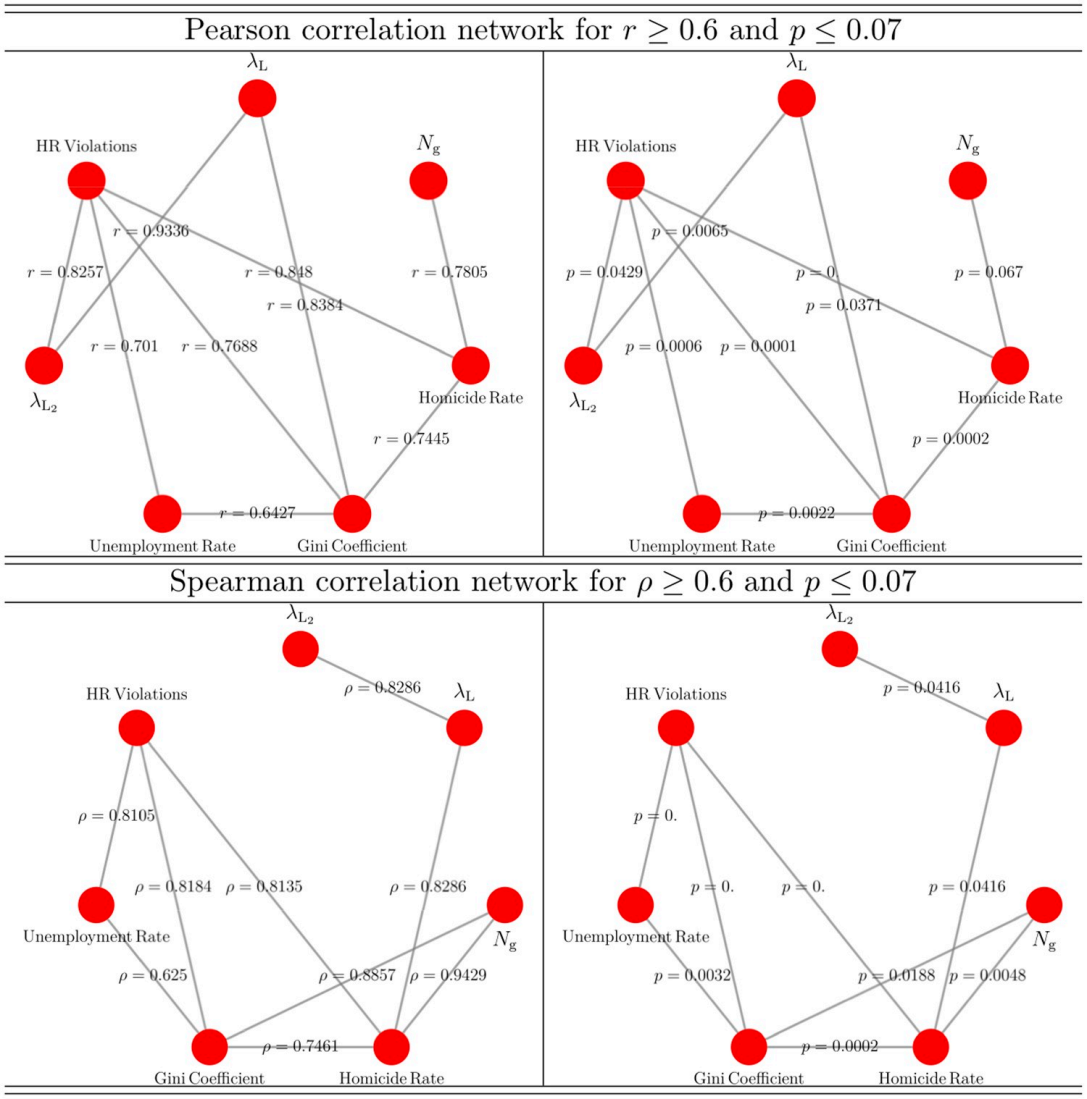
Linear and non-linear correlation networks of the number of Human Rights Violations (HR Violations), Homicide Rate (HR) per 100,000 inhabitants, Unemployment Rate, Gini Coefficient, number of gangs *N*_g_ and the leading λ_L_ and second leading $\lambda_{L_{2}}$ eigenvalues of the adjacency matrix. For the same correlation network, left panels presents the *r*, *ρ*-values and right panels do for *p*-values. Only correlations satisfying *r*, *ρ* ≥ 0.6 and *p* ≤ 0.07 were considered.

Fig 5 presents the network of linear (top panel) and non-linear (bottom panel) correlations of the confrontation network parameters with the number of Human Rights Violations (HR Violations), Homicide Rate (HR) per 100,000 inhabitants, Unemployment Rate and the Gini Coefficient. Fig 5 shows that non-linear correlations are stronger than linear ones. Specifically, note that the non-linear correlation network remains fully connected for *p* ≤ 0.05 whereas for that value of *p*, *N*_g_ is left as an isolated node in the linear correlation network. However, the fact that each network presents correlations between different variables suggests that the correlation analysis should consider both type of correlations complementarily rather than independently. Thus, Fig 5 shows that the leading eigenvalue λ_L_ of the confrontation network linearly correlates with the Gini Coefficient and is non-linearly correlated with the Homicide Rate, the second leading $\lambda_{L_{2}}$ linearly correlates with the number of Human Right Violations whereas *N*_g_ has a weak linear correlation but a strong non-linear correlation with the Homicide Rate and the Gini Coefficient.

The correlation between the number of gangs and the Homicide Rate directly follows from the armed-domination strategy of paramilitary groups in the city: gangs in opposition and not subordinated to the hegemonic paramilitary structures were annihilated or decimated; thus, increasing the number of homicides in the city. In the framework of the Frobenius-Perron theorem, λ_L_ is interpreted as the rate at which the number of confrontations increases or decreases and therefore of the number of homicides; this explains the correlation between λ_L_ and the Homicide Rate. On an abstract ground, gangs that are highly connected to other highly connected gangs will incur in a higher probability of cascade violence. The second leading eigenvalue $\lambda_{L_{2}}$ can be interpreted as the rate of convergence to equilibrium distribution and it is associated to the mixing time of the network; this explains why it correlates with HR Violations, which not only incorporates assassination of gang members. Fig 5 thus allows to conclude that the structure of the confrontation network, the socio-economic variables and violence indexes in the city are certainly correlated.

### BLV model for the confrontation network

**Fig 6:**
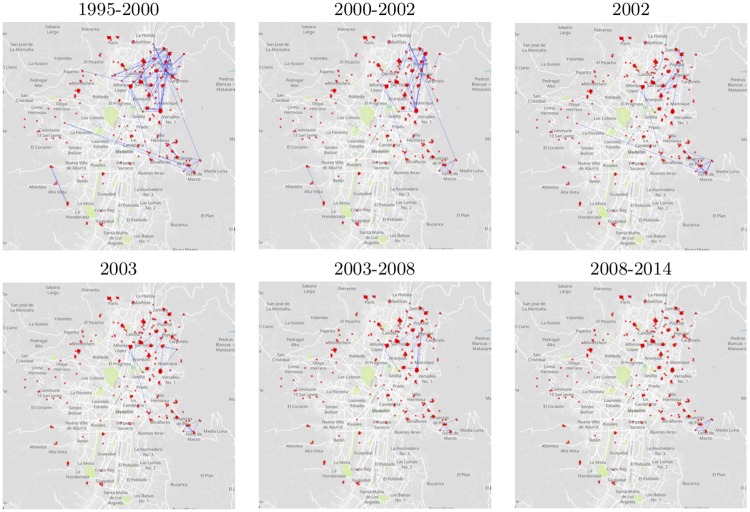
Gang confrontation network in Medellin. The localization of the gangs is presented as a red dot in the map of Medellin and the enmity ties are represented by blue lines. The background map was built using Mapbox.

**Fig 7:**
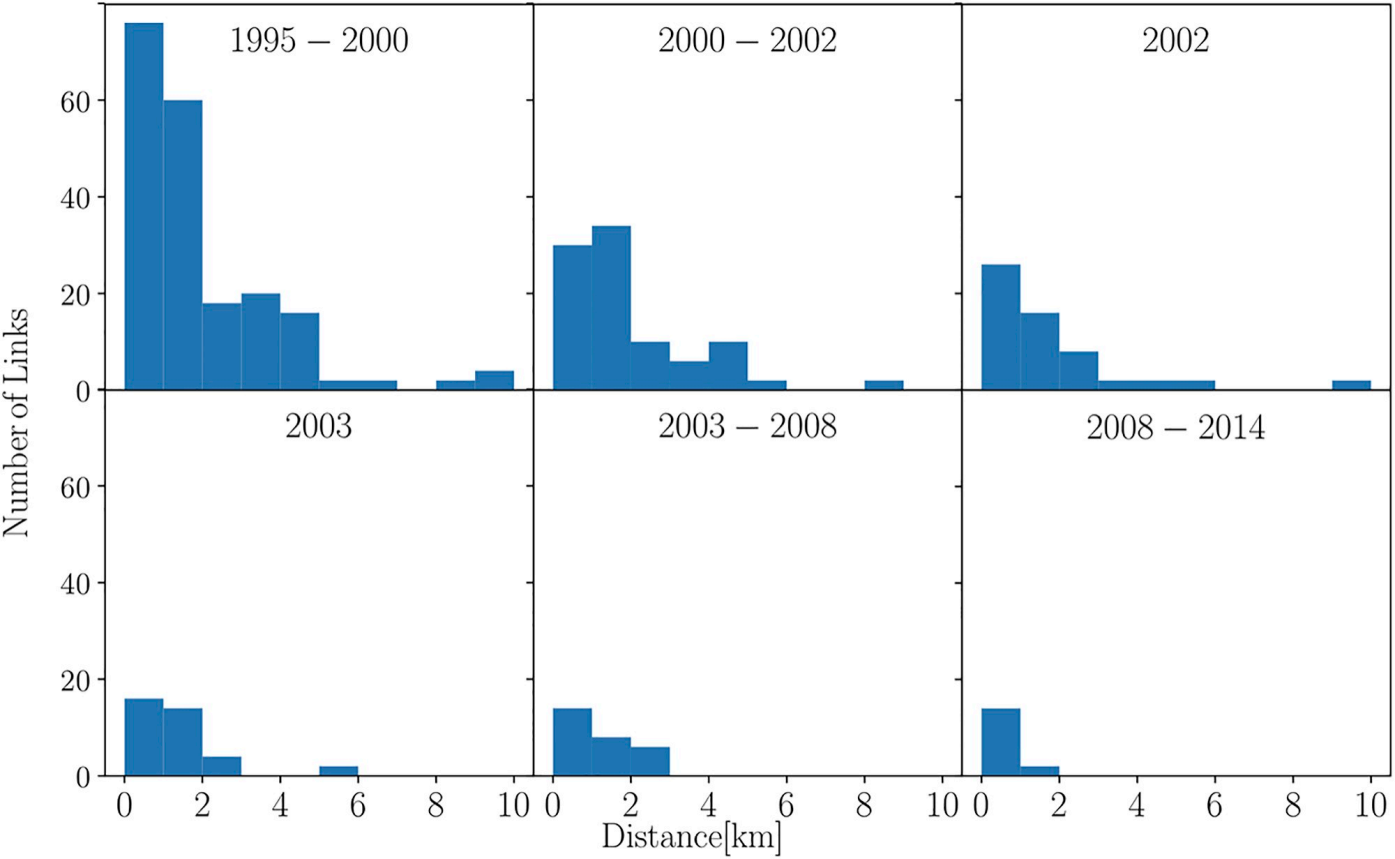
Distribution of links based on the geographic distance between nodes.

Fig 6 depicts the geographic location of gangs in the city by red dots and the hostility ties by blue lines between them. The network is highly space-correlated, i.e., the ties among the nodes are mainly between nearby nodes instead of distant nodes. This is also supported by the distribution of links based on the geographic distance between nodes presented in Fig 7. Therefore, the geolocalization of gangs is a significantly important factor to deeply understand their behaviour in the network. This is particularly significant since it points to the fact that the conflict relations of gangs in Medellin are in great proportion related to territory control, confirming the hypothesis previously proposed by other authors [2, 35–38].

**Fig 8:**
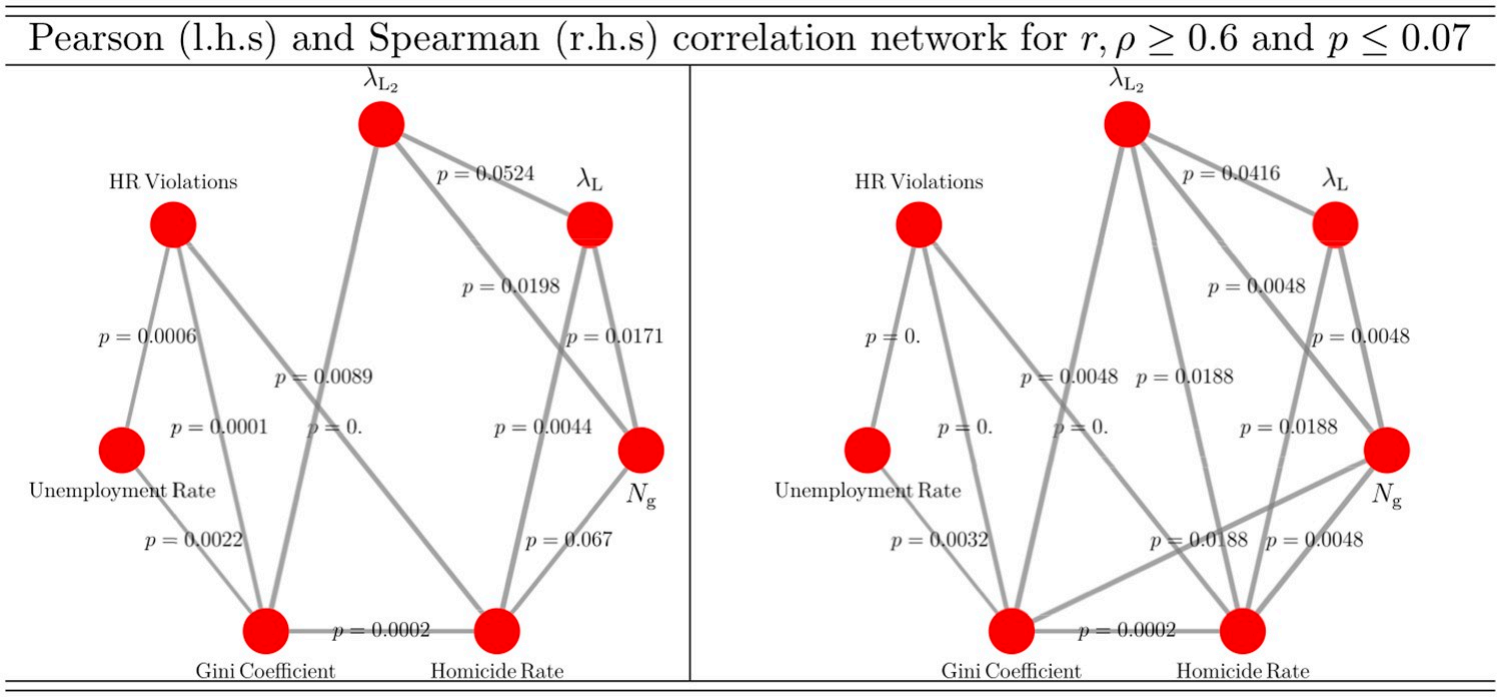
Linear and non-linear correlation networks of the number of Human Rights Violations (HR Violations), Homicide Rate (HR) per 100,000 inhabitants, Unemployment Rate, Gini Coefficient, number of gangs *N*_g_ and the leading λ_L_ and second leading $\lambda_{L_{2}}$ eigenvalues of the adjacency matrix. Edges’ thickness is proportional to *r*, *ρ* coefficients (the thickest edge corresponds to the *r* = 0.943 correlation between the Gini Coefficient and *N*_*g*_ whereas the thinest edge corresponds to *r* = 0.625 correlation between the Gini Coefficient and the Unemployment Rate. Only correlations satisfying *r*, *ρ* ≥ 0.6 and *p* ≤ 0.07 were considered.

Therefore, the geolocalisation of gangs is a significantly important factor to deeply understand their behaviour in the network and motivates the usage of spatial network models [39]. Specifically, the fact that gangs operate under cost-benefit principles suggests that the BLV formalism can be a sensible approach to model Medellin’s gang phenomenon; however, the non-stationary character of Medellin’s violence may undermine its applicability. Since the number of nodes for which confrontation information was available (blue lines in Fig 6) decreases in the geo-referenced network and the graph is considered unweighted (see above), then it is instrumental to recalculate the correlation network, results are presented in Fig 8 for linear (l.h.s panel) and non-linear (r.h.s panel) correlation measures.

In contrast to Figs 5 and 8 shows that the context variables (HR Violations, Homicide Rate, Unemployment Rate and Gini Coefficient) and confrontation network properties (*N*_g_, λ_L_ and $\lambda_{L_{2}}$) aggregate in well defined fully-connected-closed-networks. For linear correlations, “interaction” between closed networks is provided by the correlation between λ_L_ and $\lambda_{L_{2}}$ with the Homicide Rate and Gini Coefficient, respectively. For non-linear correlations, “interaction” between closed networks is provided by the additional correlation between $\lambda_{L_{2}}$ and Homicide Rate. Despite the differences between the full and the geo-referenced confrontation-networks, and therefore of the particular correlations between variables, the strong non-linear correlation between λ_L_ and the Homicide Rate is common to both confrontation-networks. This is a robust and key finding of the present work.

#### BLV-predicted adjacency confrontation matrix

**Fig 9:**
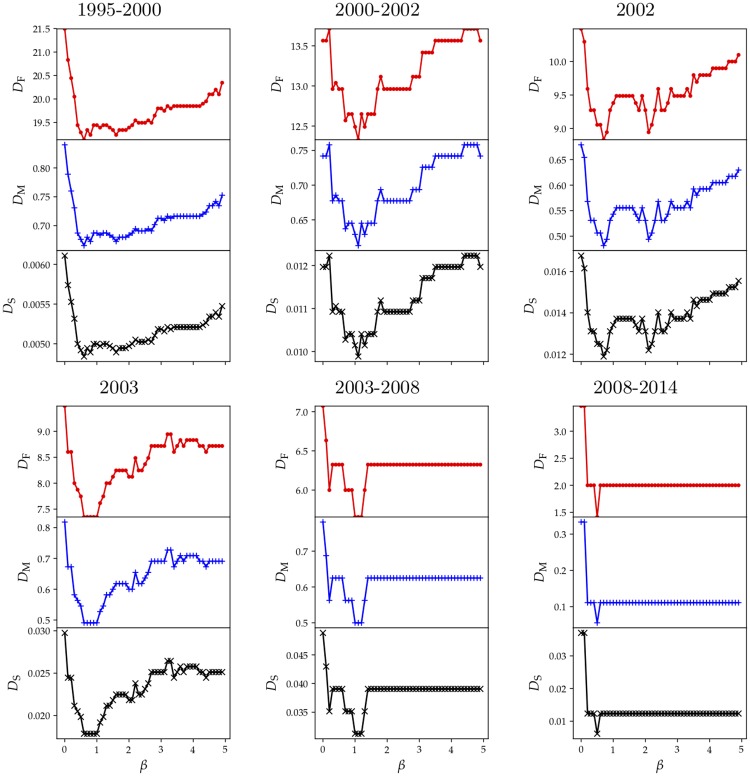
Matrix distance *D*_F_, *D*_M_ and *D*_S_ as a function of *β* parameter. For each period of time, optimal the value corresponds to *β* = {0.6, 1.1, 0.7, 0.6, 1.0, 0.5}. A high value of *β* represents a short distance interaction and conversely, a low value implies long-range interaction.

For each period of time, the values of the three distance measures defined above are shown in Fig 9. The Multiplying distance *D*_M_ is bounded: 0 ≤ *D*_M_ ≤ 1 ranging between identical matrices to matrices having orthogonal support, respectively. Since adjacency matrices considered here are not normalized, then it is not straightforward to assign a particular interpretation to a given value of the distance measures. However, note that the behavior of the three measures, as a function of *β*, is identical. Therefore, despite that high spatial character of the confrontation network in Figs 7, 6 and 9 shows that the BLV method does not accurately described the reconstructed adjacency matrix since *D*_M_ ∼ 0.5. This can be a consequence of the small dataset in the present version of the reconstructed network or, as anticipated above, an indication that the BLV may not applied to Medellin’s scenario due to the lack of stationary character of confrontations. Different alternatives to quantitatively predict the spatial embedding of the conflict are currently under study.

## Conclusions

The analysis presented in Fig 3 allows concluding that the conflict networks are resilient against removing random nodes, but weak against the targeted deletion of high–degree nodes. Figs 5 and 8 clearly show that the socio-economic variables utilized here and the confrontation network properties are moderate to highly correlated to the escalation of conflict in Medellin during the last twenty years. Particularly, the correlation among the leading eigenvalue of the adjacency matrix λ_L_ and the Homicide Rate allows to conclude that the topological structure of the network is a significant descriptor of the violence in the city. Since the leading eigenvalue is always higher than two, based on the Frobenuios-Perron theorem, it can be concluded that the conflict network does not reach stability. This can be interpreted as a measurement of the instability of the conflict network, leading to retaliation among gangs and hence manifested as an occurrence on the number of Human Rights Violations in the city and vice-versa.

Figs 7 and 6 suggest that the conflict network of gangs in Medellin is spatial. However, the BLV formalism do not accurately describe the reconstructed confrontation network and different alternatives to quantitatively predict the spatial embedding of the conflict are currently under study. To summarize, a high correlation between the structure of a gang confrontation network and the escalation of conflict in Medellin was presented and that the territory control mechanism is a main driven force of the conflict among the gangs in Medellin.

## Supporting information

S1 Table Socio-economic data. Dataset of socio-economic variables and matrix properties.
(XLSX)

S1 Script Mathematica 11.0 Script. This Mathematica 11.0 script generates the results presented in Figs 2 and 3 and Tables 1 and 2 of the manuscript.
(NB) Click here for additional data file.

S2 Script Mathematica 11.0 Script. This Mathematica 11.0 script generates the results in Figs 5 and 8 of the manuscript.
(NB) Click here for additional data file.

S1 DataFile Mathematica 11.0 Data File. Contains the node name of each gang.
(MX) Click here for additional data file.

S2 DataFile Mathematica 11.0 Data File. Contains the edges of the confrontation network before the Bloque Metro period.
(MX) Click here for additional data file.

S3 DataFile Mathematica 11.0 Data File. Contains the edges of the confrontation network during the Bloque Metro period.
(MX) Click here for additional data file.

S4 DataFile Mathematica 11.0 Data File. Contains the edges of the confrontation network during the Bloque Metro and Bloque Cacique Nutibara Confrontation period.
(MX) Click here for additional data file.

S5 DataFile Mathematica 11.0 Data File. Contains the edges of the confrontation network during the Bloque Cacique Nutibara period.
(MX) Click here for additional data file.

S6 DataFile Mathematica 11.0 Data File. Contains the edges of the confrontation network during the Bloque Heroes de Granada period.
(MX) Click here for additional data file.

S7 DataFile Mathematica 11.0 Data File. Contains the edges of the confrontation network during the DDR period.
(MX) Click here for additional data file.
